# Catheter Tip Migration in Female Patients With Breast Cancer: A Retrospective Comparative Study of Right- and Left-Sided Chest Ports

**DOI:** 10.1155/tbj/7358397

**Published:** 2024-12-21

**Authors:** Alexander T. O'Mahony, Aidan Coffey, Patrick W. O'Regan, Emily Walsh, Brian Carey, James Ryan, Niamh Dorney, Owen J. O'Connor, Jack Gleeson, Stephen P. Power

**Affiliations:** ^1^Department of Radiology, Cork University Hospital, Cork, Ireland; ^2^Department of Radiology, Mercy University Hospital, Cork, Ireland; ^3^Department of Radiology, School of Medicine, University College Cork, Cork, Ireland; ^4^Department of Medicine, School of Medicine, University College Cork, Cork, Ireland; ^5^Department of Oncology, Cork University Hospital, Cork, Ireland; ^6^Department of Medicine, Cork University Hospital, Cork, Ireland

**Keywords:** catheter tip migration significance, left-sided chest port, standard imaging for catheter tip position confirmation

## Abstract

**Introduction:** Chest ports are typically inserted via the right internal jugular vein with the left side being utilized in certain patient populations. The purpose of this study was to evaluate the dynamic position of the chest port and catheter tip, comparing a demographically matched cohort of female breast cancer patients with right- or left-sided chest ports.

**Methods:** 142 female patients with breast cancer requiring chest port insertion for chemotherapy and imaging confirming catheter tip position initially with supine fluoroscopy and follow-up with erect chest radiography over a 5-year period were identified. Data points analyzed were catheter tip-to-carina distance and the distance from the port to the ipsilateral infraclavicular border. Intraprocedural measurements were taken in the supine position during chest port insertion and compared with follow-up erect chest radiography. The catheter tip position was also allocated a zone within the venous system on both image sets to assess for significant retraction to a more proximal zone in the erect position. Imaging within 12-months of chest port insertion was also screened to identify port-related complications.

**Results:** The whole cohort showed significant retraction of the catheter tip (cephalad) (*p* < 0.001) and protraction of the port (caudal) (*p* < 0.001). The median tip-to-carina distance decreased from 38.3 mm to 28.6 mm and the port-to-clavicle distance increased from 31.3 mm to 64.6 mm. Right-sided chest ports had increased tip-to-catheter retraction (15 mm) compared with left-sided (6.9 mm) (*p*=0.310). A complication was identified in 8.5% of the right-sided and 11% of the left-sided ports. Zone migration was significantly associated with the occurrence of a complication in left-sided ports (*p*=0.023).

**Conclusion:** When assessing chest port catheter tip position between supine and erect radiographic studies in female patients with breast cancer, retraction cephalad will occur and this is more apparent in right-sided ports. Change in catheter tip position was not associated with a significant increase in complication rate unless it occurred in left-sided ports where zone migration was evident.

## 1. Introduction

Chest ports are frequently used in patients requiring chemotherapeutic agents. In general, they are safe with relatively low complication rates (1.3–7.2%) [1, 2]. Complications can include infection, thrombosis, malposition, migration, and obstruction [3]. Correct placement and positioning of the catheter tip at the time of insertion are associated with a decreased risk of significant migration, mechanical obstruction, and thrombosis [4, 5]. However, the catheter tip position is dynamic and as a patient moves from supine to standing, the catheter tip retracts/migrates cephalad. This has implications when considering the method of tip position confirmation, i.e., patient supine at fluoroscopy vs. erect at standard chest radiograph postinsertion.

Tip retraction in the upright position is more pronounced in females and in those with higher body mass index (BMI) due to the downward traction of breast or adipose tissue [6]. This should be taken into consideration when confirming the tip position. The optimal position of a central venous catheter tip is debated but has been defined as a zone in the area of the proximal right atrium/cavoatrial junction/distal superior vena cava (SVC) by the Association of Anesthetists of Great Britain and Ireland (AAGBI)/European Society for Clinical Nutrition and Metabolism (ESPEN)/British Committee for the Standards in Hematology (BCSH), respectively [7]. The right internal jugular vein (IJV) is preferably used as the venous access site for central venous access due to the vertical course to the right atrium [8, 9]. However, relative contraindications may exist to a right-sided approach such as intended radiation field, site of breast cancer, distorted local anatomy, and thrombus in the right IJV. This can warrant a left-sided approach for chest port insertion.

The literature is equivocal as to whether a left-sided approach has increased the incidence of complications with a paucity of information regarding the relationship between chest port complications and the degree of catheter tip migration. A previous study in oncology patients found that mechanical port complications were independent of the chest port insertion side but an association with catheter tip position was not analyzed [10].

The present study aimed to evaluate the difference in catheter tip position on supine fluoroscopy and erect chest radiography between right- and left-sided chest ports in female patients with breast cancer. A further objective was to compare catheter-related complications in these two groups in the context of tip location and relative to the degree of retraction within 12 months of port insertion.

## 2. Methods

### 2.1. Study Design and Outline

A retrospective evaluation of imaging in female breast cancer patients was performed. An analysis of chest port catheter tip and port migration between initial supine fluoroscopic imaging and follow-up erect X-ray imaging was performed comparing outcomes between right- and left-sided chest ports.

### 2.2. Ethical Approval

Institutional review board ethical approval was granted in April 2021 (reference number: ECM 4 (ii) 11/05/2021).

### 2.3. Patient Inclusion

Female patients with breast cancer referred for chest port insertion at a single institution over a 5-year period (January 2016–December 2020) were identified. Identification involved a distinct field search on the institutional picture archive and communication system (PACS). Fields included “female” and “chest port insertion” within the specified study period. Each clinical request was screened for the indication of chest port insertion. If no discernible indication could be identified, patient medical records (PMRs) were reviewed. Only patients requiring insertion for breast cancer treatment were included. The BMI of each patient at the time of chest port insertion was also retrieved from the PMR.

### 2.4. Chest Port Insertion

Chest ports (Polysite implantable port, Perouse Medical, Route du Manoir, 60,173 Ivry Le Temple, France) were all inserted or supervised by five fellowship-trained interventional radiologists using a standardized institutional protocol, aiming to place the catheter tip in the area of the proximal right atrium/cavoatrial junction/distal SVC. In patients being treated for right-sided breast carcinoma, the left side was used for access. Ultrasound was used for initial venous access in each patient. The catheter tip position was confirmed via fluoroscopy at the end of each procedure.

### 2.5. Data Retrieval

Relevant identified studies were retrieved on the PACS. Fluoroscopic images were taken immediately postport insertion as per the institution protocol to clarify the catheter tip position. Follow-up erect chest radiographs (anterior–posterior/posterior–anterior) were analyzed to detect a difference in the tip position by direct measurement and comparison relative to the initial supine study.

### 2.6. Radiological Data Points

**Figure 1:**
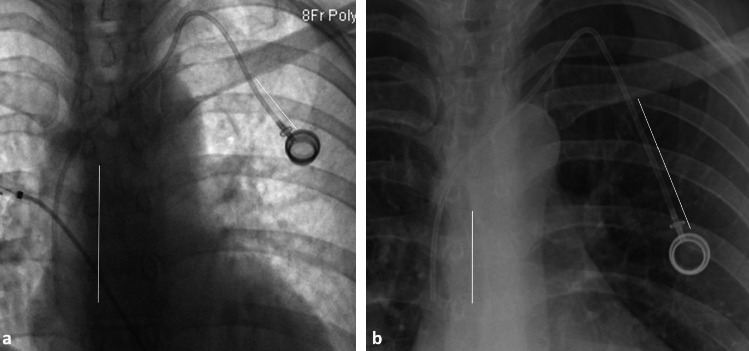
Left-sided port measurements. (a) Magnified fluoroscopic image of initial left-sided port insertion demonstrating typical measurement locations (solid white lines). (b) Magnified follow-up chest radiograph demonstrating typical measurement locations (solid white lines). This example is a 45-year-old female with a left-sided port in situ and 3-day interval between imaging. Δ catheter tip-to-carina: −20.3 mm (cephalad, retracted); Δ port: +31.6 mm (caudal, protracted); zone on fluoroscopy: upper right atrium (Zone A); zone on follow-up: distal superior vena cava (Zone A).

**Figure 2:**
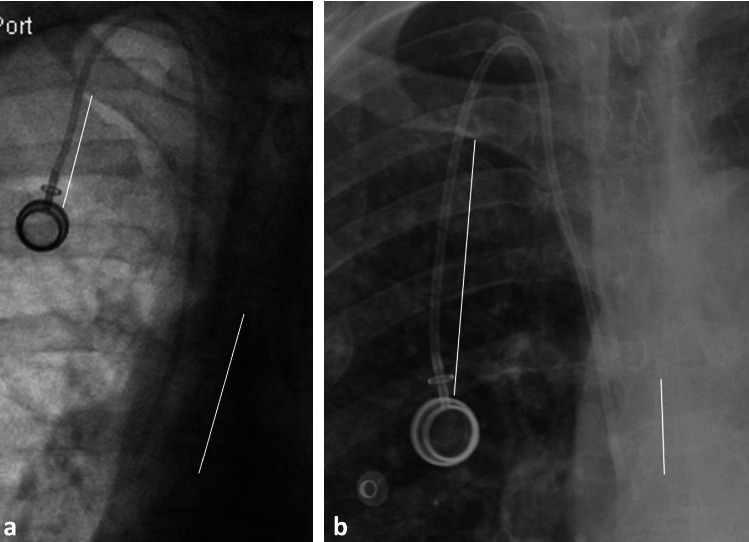
Right-sided port measurements. (a) Magnified fluoroscopic image of initial right-sided port insertion demonstrating typical measurement locations (solid white lines). (b) Magnified follow-up chest radiograph demonstrating typical measurement locations (solid white lines). This example is a 66-year-old female with a right-sided chest port in situ and a 2-month interval between imaging. Δ catheter tip-to-carina: −19 mm (cephalad, retracted); Δ port: +36 mm (caudal, protracted); zone on fluoroscopy: distal superior vena cava (Zone A); zone on follow-up: midsuperior vena cava (Zone A).

**Figure 3:**
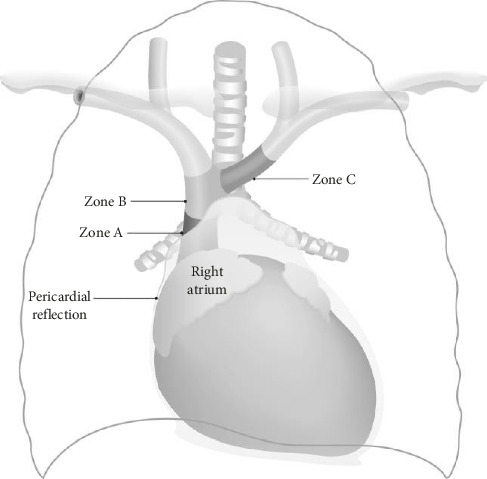
Zones of catheter tip location. Stylized anatomical figure dividing the great veins and upper right atrium (RA) into three zones (A–C), representing different areas of significance for placement of CVCs. Zone A: upper RA and lower SVC; Zone B: upper SVC and the junction of the left and right innominate veins; Zone C: left innominate vein [11].

Images were evaluated on a PACS workstation hosted by IMPAX (Agfa HealthCare, Mortsel, Belgium). Calculation of the catheter tip-to-carina distance required identification of both the carina and catheter tip on the film and a measurement calculated between these two points parallel to the adjacent spinous processes. The port-to-clavicle calculation involved measuring the point where the central line tubing exited the subcutaneous implantable device and intersected with the ipsilateral infraclavicular border (Figures 1 and 2). The position of the tip was further classified according to the venous zone. Zoning was described according to Stonelake and Bodenham (Figure 3), where Zone A is the upper right atrium and lower SVC, Zone B is the upper SVC and junction of the left and right innominate veins, and Zone C is the left innominate vein [11].

Each imaging series (fluoroscopy and erect follow-up) was measured and zoned by two readers (AC and EW) blinded to each other's results after supervision by a consultant interventional radiologist (SP) and satisfaction with the accuracy of each investigator's respective measurements.

Any further radiological imaging conducted within the 12 months of port insertion was analyzed by an initial consultant-approved clinical report and recorded if a port-related complication occurred.

### 2.7. Data Analysis

Two separate Microsoft Excel 2011 spreadsheets (Microsoft Corporation, Seattle, Washington, U.S) were generated for each investigator. Within a respective dataset, tabulation for right- and left-sided port data existed with population fields for each previously described metric as well as demographics.

### 2.8. Statistical Analyses

The intraclass correlation coefficient (ICC) was used to assess for interobserver bias between measurements, mean, and standard deviation (Gaussian distribution), or median with upper and lower interquartile ranges (non-Gaussian) of measurements was calculated and tabulated. Either t-testing or Wilcoxon signed-rank testing was used to assess for significance depending on normality for scaled data when assessing between 2 variables. Analysis of variance (ANOVA) or Kruskal–Wallis test was utilized when assessing for more than 2 groups/independent samples. Categorical data were compared using chi-square or Fisher's exact test. Direct correlation between scaled data was assessed by either Pearson or Spearman coefficients. A *p* value of < 0.05 was considered significant, and all *p* values reported were two-tailed. Data are presented as mean ± SD unless otherwise specified in the text.

## 3. Results

### 3.1. Demographics

142 patients were identified over the 5-year period with imaging confirming catheter tip position initially with supine fluoroscopy and follow-up with erect chest radiography. 91 patients had a right-sided chest port inserted with the remainder having left-sided chest ports inserted. A mean age of 53.8 ± 11.9 years and a BMI of 29.1 ± 6.6 kg/m^2^ were recorded.

The median interval between index (fluoroscopy) and follow-up (erect chest radiography) was 63 days, with the lowest and highest quartiles at 18.5 and 139 days, respectively. Age, BMI, and follow-up interval were all matched between right and left groups (age *p*=0.899, BMI *p*=0.434, and interval *p*=0.568).

All the patients' medical records and available follow-up imaging were reviewed for up to 12 months to identify port-related complications. Twenty-one (15%) complications were identified which included 7 port infections, 6 fibrin sheaths with obstruction of the catheter tip, 6 venous thromboses, and 2 cases of port rotation.

### 3.2. Interrater Reliability

The ICC reported all R-values of > 0.8 and > 0.9 for port-to-clavicle measurements between two independent observers (Table 1).

### 3.3. Entire Group: BMI

BMIs were provided for 118 of the 142 patients. As per the World Health Organization (WHO) classification, 39 were obese, 49 overweight, 27 normal, and 3 underweight. Static BMI did not correlate with a change in catheter tip position (*p*=0.171), although a positive but low correlation existed with a change in chest port position (*p* < 0.001, *R* = 0.46). The mean change in chest port position increased with the WHO BMI category (*p* < 0.001), with those who were overweight or obese displaying greater values in a linear direction (Table 2).

### 3.4. Entire Group: Migration

Between studies, the catheter tip migrated by a median of −12.1 mm (IQR: +4.2 mm to −26.6 mm) (*p* < 0.001), i.e., cephalad migration/tip retraction. The port-to-clavicle distance increased by a median of +31.4 mm (IQR: +22 mm to +48.7 mm) over the interval (*p* < 0.001), i.e., caudal migration/downward protraction (Table 3).

### 3.5. Dichotomized Groups: Right vs. Left

Right-sided chest port catheter tips retracted by a median of −15 mm (IQR: +3.8 mm to −28.8 mm) with a median port protraction of 34.3 mm (IQR: +19.1 mm–49.8 mm). Left-sided chest ports demonstrated tip retraction by a median of −6.9 mm (IQR: +4.4 mm to −24.3 mm) and a port protraction of +29.6 mm (IQR: +24 mm to +42.5 mm), although neither achieved significance when directly compared (*p*=0.310 and *p*=0.6013, respectively).

### 3.6. Dichotomized Groups: Outcome

Nineteen right-sided ports required further imaging for suspected chest port–related complications, 12 (8.5%) of which were confirmed: five infections, four venous thromboses, two port rotations, and one fibrin sheath were identified.

Thirteen left-sided insertions required further imaging with nine (6%) confirmed complications: five fibrin sheaths with catheter tip obstruction, two venous thromboses, and two infections were identified. Complication rates were not associated with the side of port insertion (*p*=0.571).

Patients with a right-sided chest port and associated complications had a median catheter tip migration of +1.3 mm, i.e., port protraction vs. a retraction of −16.5 mm in those without complication (*p*=0.481). In the left-sided cohort, a median catheter tip retraction of −11.9 mm was demonstrated in those with complications vs. −6.9 mm in those without complications (*p*=0.586) (Table 4). The intergroup comparison, i.e., complications in left-sided chest ports vs. right-sided chest ports was not significant (*p*=0.345).

In 26 patients, the catheter tip retracted to a more proximal zone on follow-up imaging. This occurred in 18 right-sided chest ports and 8 left-sided chest ports (*p*=0.545). The majority of catheter tip migration was from Zone A to Zone B with 14 (78%) of the right-sided ports and 7 (88%) of the left-sided ports demonstrating this migration. Zone migration was not associated with complications for the entire group (*p*=0.112). However, migration to a proximal zone was significantly associated with complications in left-sided chest ports (*p*=0.023). A right-sided association was not significant (*p*=0.985) (Table 5).

## 4. Discussion

This study assessed the dynamic position of the chest port catheter tip and port reservoir position between a demographically matched cohort of female patients requiring chest port insertion for venous access in breast cancer treatment. As the position of the chest port catheter tip and port reservoir can vary dramatically between supine and erect chest imaging, the outcome of interest was a like-for-like comparison between left and right chest ports along with risk factors for adverse outcome, i.e., chest port–related complications.

Tip migration is undesirable for two reasons in cancer patients, the first being that smaller caliber vessels respond adversely to cytotoxic agents resulting in vessel irritation, local phlebitis, and potentially thrombosis [12]. The second is that mechanical complications such as obstruction requiring chest port removal are more likely, ensuing both a clinical and resource burden [13].

In our analysis, there was evidence of tip retraction when assessing the entire population (−12.1 mm, IQR: +4.2 mm to −26.6 mm, *p* < 0.001), which is in agreement with multiple other studies [6, 11, 13, 14]. As tip position confirmation was with supine fluoroscopic imaging and follow-up with erect chest radiography, this apparent migration is to be expected [6]. The clinical importance of this is that chest ports are typically accessed in the upright position (with the patient sitting or in the semirecumbent position), therefore, the erect chest radiograph is of most relevance when considering the risk of the complications described.

On like-for-like comparison between right- and left-sided chest ports, catheter tip migration was larger in right-sided chest ports but this was not statistically significant (Δ median: −15 mm (right) vs. −6.9 mm (left), *p*=0.310).

A similar study, albeit assessing tip position on index chest port insertion and follow-up chest port removal with fluoroscopy, indicated results of a contrary kind, where left-sided port catheter tips retracted significantly more than their right-sided counterparts on t-testing with *a* > 20 mm difference and increased incidence of dysfunction for both if migratory [15]. This contrary result may be related to these authors measuring the length of retraction in the supine position both at the time of insertion and removal [15]. Given the unreported matching of the right and left subgroups and large standard deviations in the reported metrics, the applicability of these findings is uncertain [15].

Our study demonstrated that catheter tip retraction in the left-sided chest port cohort was associated with the development of a related line complication. In this group, migration occurred by a median of −11.9 mm (retraction) with the interquartile range 25^th^ and 75^th^ both being negative, i.e., 50% of patients who developed complications demonstrated tip retraction. Those without a complication had a median retraction of −6.9 mm with the interquartile range crossing the 0 point. This supports findings from prior studies [15].

The identified complication rate was 15% amongst our cohort, which is somewhat higher than that reported in the previous literature [1–3]. Possible causes for the slightly higher rate of complications in our cohort were the mean BMI of 29.1 kg/m^2^ and oncologic cohort factors which are known to be associated with increased complication rates [16]. Complications were more likely in patients with left-sided chest ports when retraction of the tip resulted in a proximal zone migration (*p*=0.023). Retraction between zones occurred in 8 patients with left-sided chest ports, with three of these patients having chest port–related complications identified. This finding was not identified in patients with right-sided chest ports. Consequently, operators may consider increasing the length of port catheters in left-sided port insertions to potentially mitigate port tip retraction–related complications.

The limitations of this study include its retrospective single-center nature. The specific patient cohort of female patients with breast cancer, small sample size, and attrition due to incomplete data limit the applicability of the results in a broader sense. However, given the matched demographics between groups and appropriate statistical testing according to normality distribution, a direct comparison is enabled. In addition, the high ICC evident between blinded readers attests to the accuracy of the metrics evaluated.

Another limitation is that as imaging was conducted by different methods, i.e., supine fluoroscopy vs. erect chest radiography, the effects of magnification as well as parallax may affect the results reported for the entire group but should not affect the intragroup comparisons as these essentially are analyzing vectors (Δ values) for significance in both groups.

## 5. Conclusion

Catheter tip migration cephalad is inevitable between supine and erect X-ray studies. This is potentially more pronounced in those with right-sided chest ports. Radiologically diagnosed chest port–related complications are associated with zone migration in left-sided chest port catheter tips in female patients with breast cancer. Operators may consider placing port catheter tips more caudally in female patients with breast cancer when a chest port is placed on the left side to potentially reduce zone migration and subsequent complications.

## Figures and Tables

**Table 1:** Interrater reliability between two observers with R-coefficient for measurements.

Anatomic measurement	*R*-value	*p* value
Tip-to-carina index	0.879	< 0.001
Tip-to-carina follow-up	0.896	< 0.001
Port-to-clavicle index	0.959	< 0.001
Port-to-clavicle follow-up	0.917	< 0.001

**Table 2:** Change in chest port-to-clavicle distance (*n* = 118).

BMI category	Number	Mean (mm)
< 18.5 underweight	3	31.8 ± 12.2
18.5–25 normal	27	24.9 ± 11.7
25–30 overweight	49	33.0 ± 16.5
> 30 obese	39	41.2 ± 17.8

**Table 3:** Catheter tip and port reservoir migration for the entire group (*n* = 142).

Quartiles	Index (mm)	Follow-up (mm)	Δ (mm)	*p* value
*Tip-to-carina (mm)*				
25^th^	26.8	11.9	+4.2	< 0.001
Median	38.3	28.6	−12.1	
75^th^	50.3	41.9	−26.6	

*Port-to-clavicle (mm)*				
25^th^	22.9	51.5	+22	< 0.001
Median	31.3	64.6	+31.4	
75^th^	42.6	81.9	+48.7	

**Table 4:** Right vs. left catheter tip and port reservoir migration.

	Complication	Median	IQR: low	IQR: high	*p* value
*Right*					
ΔTip-to-carina	Yes	+1.3	+8.6	−36.6	0.481
	No	−16.5	+2.1	−28.8	
ΔPort-to-clavicle	Yes	+24.1	+12	+47.9	0.234
	No	+34.4	+21.9	+49.8	

*Left*					
ΔTip-to-carina	Yes	−11.9	−1.2	−28.5	0.586
	No	−6.9	+5.6	−25.6	
ΔPort-to-clavicle	Yes	+28.1	+22.7	+36	0.568
	No	+29.6	+23.6	+44.2	

**Table 5:** 2 × 2 tables demonstrating complication and zone migration of catheter tip.

**Right-sided chest port**					**Left-sided chest port**				
		**Zone migration**		**Total**			**Zone migration**		**Total**
		**Yes**	**No**				**Yes**	**No**	

Complication	*Yes*	2	8	10	Complication	*Yes*	3	2	5
	*No*	16	65	81		*No*	5	41	46
Total		18	73	91	Total		8	43	51
*P* value				0.985	*P* value				0.023

## Data Availability

Data are available on reasonable request from the corresponding author.
